# A bone-conserving revision stem for unstable intertrochanteric fractures of the geriatric osteoporotic population

**DOI:** 10.1186/s42836-022-00151-6

**Published:** 2022-11-05

**Authors:** Mengcun Chen, Jinlong Wang, Adnan N. Cheema, Shuhua Yang, Xianzhe Liu

**Affiliations:** 1 Department of Orthopedics, Wuhan Union Hospital, Tongji Medical College, Huazhong University of Science and Technology, Wuhan, 430022 China; 2 Department of Orthopaedic Surgery, Mayo Clinic, 200 First St NW, Rochester, MN 55901 USA

**Keywords:** Hip, Unstable intertrochanteric fracture, Osteoporosis, Bipolar hemiarthroplasty, Cementless stem, Fully hydroxyapatite coating

## Abstract

**Purpose:**

Primary hemiarthroplasty is gaining popularity for the treatment of unstable intertrochanteric fractures in geriatric patients with severe osteoporosis. This study evaluated early clinical and radiographic outcomes by using a bone-conserving revision stem for unstable intertrochanteric fractures in the geriatric osteoporotic population.

**Methods:**

A retrospective study involving 31 patients with unstable intertrochanteric fractures was conducted. The patients were aged 82.1 years on average. All patients underwent primary hemiarthroplasty using bone-conserving, fully porous-coated revision stem. The operative time, intraoperative blood loss, length of hospitalization, and need for blood transfusion were noted during the hospital stay. Postoperative complications, including dislocations, deep venous thrombosis, infections, peri-prosthetic fractures, and frontal thigh pain were also recorded. Koval's category was used to quantify activity level, and Harris hip score (HHS) was used for functional assessment. Radiographic outcomes, including osteolysis, bone ingrowth, subsidence of the femoral component, lower limb length discrepancy, and heterotopic ossification, were collected at each follow-up.

**Results:**

The 31 patients were followed for an average time of 23 months postoperatively. The average operative time lasted for 74.2 min, while the mean intraoperative blood loss was 200.1 ml, with an average hemoglobin decrease of 11.1 g/L after the procedure. The mean visual analog scale (VAS) score for pain dropped from 7.4 preoperatively to 2.4 at the 4-week follow-up. At the latest follow-up, the mean Harris hip score was 82.1, and the VAS was 1.7. No intraoperative or postoperative peri-prosthetic fractures were noted. Postoperative complications included one case of thrombosis formation in the posterior tibial vein and one case of congestive heart failure. Both patients were discharged uneventfully after treatment. Radiographically, none of the hips had evidence of stem loosening or osteolysis. Within the follow-up period of 23 months, the mortality rate was 3.2% (1/31), and no revision surgeries were required.

**Conclusion:**

Primary hemiarthroplasty using a bone-conserving, cementless revision stem could serve as a reliable alternative for the treatment of unstable intertrochanteric fractures in the geriatric population with osteoporosis.

## Introduction

Hip fracture is a common occurrence and afflicts 1.6 million people annually across the globe. It is projected to affect 6.3 million worldwide by 2050 [1]. In the geriatric population, surgical management of intertrochanteric (ITC) fractures remains a challenge due to co-morbidities, poor bone quality, and fracture instability [2]. Clinically, osteosynthesis with internal fixation is the most commonly used modality for stable ITC fractures and yields satisfactory outcomes [3]. However, the utility of internal fixation has been questioned in the cases of apparent fracture instability and severe osteoporosis [4, 5]. Complications following internal fixation of ITC fractures include nonunion, malunion, fracture collapse with intra-articular screw migration, and post-traumatic osteoarthritis, resulting in significant loss of mobility and added burden on already-stressed medical resources [4–6].

Joint arthroplasty is generally accepted as a salvage option for failed internal fixation [2, 4]. Nonetheless, the conversion procedure can be fraught with complications [4]. Furthermore, geriatric patients tend to have many comorbidities, which can preclude a second operation [2]. Recently, there has been a trend to treat unstable intertrochanteric fractures in geriatric patients with hemiarthroplasty, which has shown encouraging early and mid-term outcomes [7–9]. However, joint arthroplasty can result in prolonged surgical time and increased intraoperative blood loss [2]. Additionally, poor press-fit of the stem and failure of bony on-growth due to trochanter fracture and osteoporosis still pose concerns [10].

Cementless, bone-conserving, fully-hydroxyapatite-coated femoral revision stems have been widely used in revision hip arthroplasties [11]. These familiar stems can potentially be employed in the ITC fracture setting to minimize iatrogenic bone loss and reduce surgical time [12]. The initial stability can be achieved at the metaphyseal junction, and the hydroxyapatite coating can provide an optimal osseointegration surface for long-term stability [12, 13]. However, few studies examined the use of revision femoral stems in the management of geriatric, osteoporotic, and unstable ITC fractures. Therefore, the objective of the current study was to evaluate the early clinical and radiographic outcomes of revision femoral stems in primary hemiarthroplasty for the treatment of unstable ITC fractures in the geriatric osteoporotic population.

## Patients and methods

### Patient demographics

The study was approved by the Ethics Committee of Wuhan Union Hospital (2019-S1171) and signed consent forms were acquired from all the patients involved. From July 2017 to April 2018, 31 patients (31 hips involved) who had undergone primary hemiarthroplasty for ITC fractures were retrospectively reviewed. All patients admitted were comprehensively evaluated by a Multiple Disciplinary Team (MDT) consisting of a trauma surgeon, a joint surgeon, an intensive care unit physician, an anesthesiologist, and a physical therapist. The MDT then developed a personalized treatment plan for each patient based on the fracture pattern, bone stock, pre-injury ambulatory status and comorbidities. Inclusion criteria for receiving primary hemiarthroplasty were as follows: (1) Unstable ITC fractures (three parts or more, and a loss of posteromedial cortical buttress), (2) Age of 75 years or older with a preoperative diagnosis of osteoporosis, (3) Patients who could ambulate independently with or without crutches (Koval's grade I–V) before injury [14], and (4) being scored Class I–III on the scale of the American Society of Anesthesiologist (ASA). Preoperative demographic data are summarized in Table 1.

**Table 1:** Demographics of patients underwent primary hemiarthroplasty

Demographic	
*Number of patients*	31
*Gender*	
*Male*	14
*Female*	17
*Average age (years)*	82.1± 4.7
*Osteoporosis (T score)*	
*Lumbar*	-3.14 ± 0.41
*Contralateral hip*	-2.96 ± 0.26
*Comorbidity*	
*Hypertension*	20 (64%)
*Diabetes*	10 (32%)
*Cerebrovascular accident*	4 (13%)
*COPD*	3 (10%)
*Auricular fibrillation*	3 (10%)
*Coronary heart disease*	2 (6%)
*Parkinson’s disease*	1 (3%)
*Chronic renal failure*	1 (3%)
*Evans-Jesen classification*	
*III*	7
*IV*	11
*V*	13
*ASA class*	
*I*	1
*II*	11
*III*	19
*Koval’s grade by pre-fracture*	
*I*	12
*II*	8
*III*	2
*IV*	5
*V*	3

*ASA* American Society of Anesthesiologist

### Implant characteristics

**Fig. 1:**
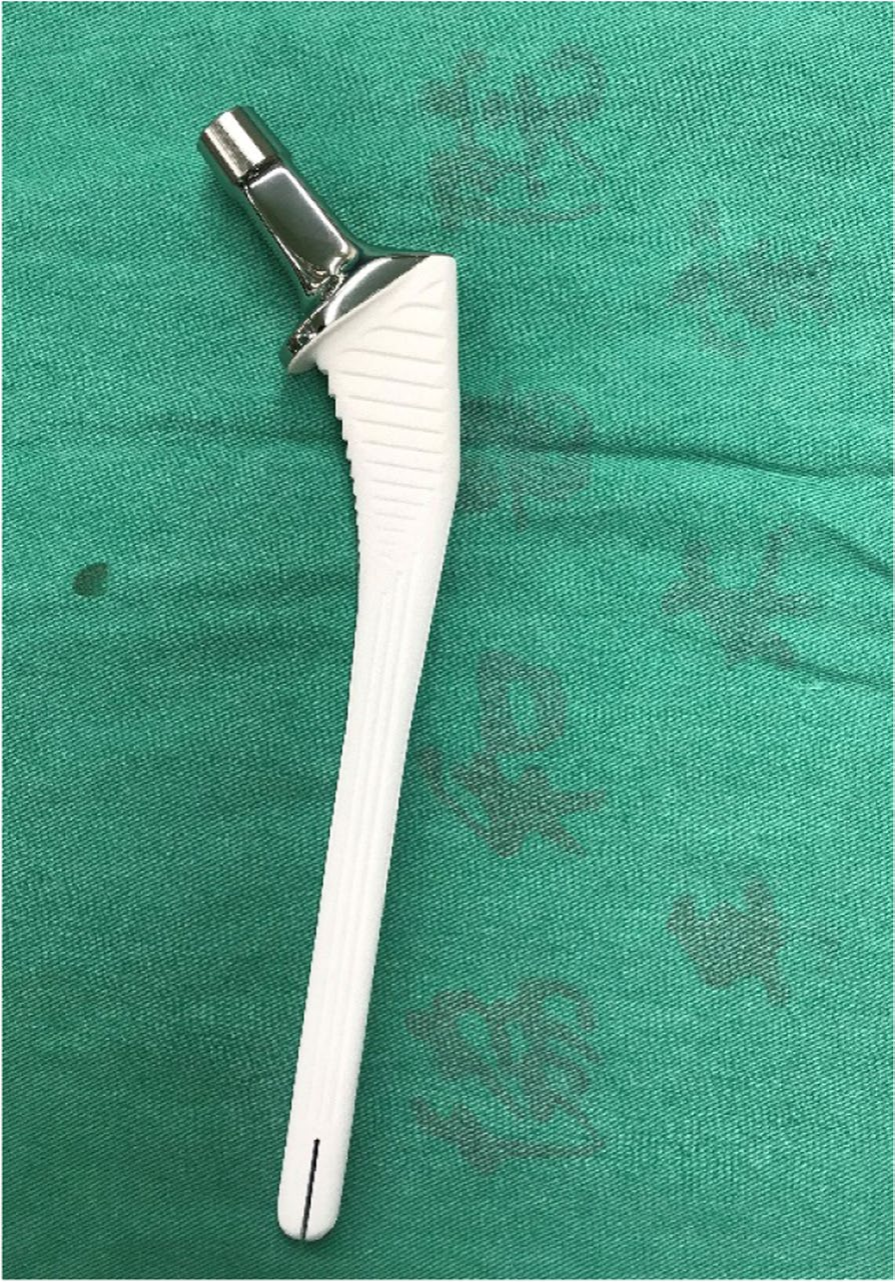
The bone-conserving revision stem is a cementless, tapered titanium stem with a collar and extensive hydroxyapatite coating on the surface

A cementless, tapered titanium femoral stem with an extensive hydroxyapatite coating was used in all cases. The design was based on the CORAIL and KAR™ Hip Systems (Depuy Synthes, New Brunswick, NJ, USA) (Fig. 1). The acetabular component used was a corresponding cobalt-chromium cup with an ultra-high molecular weight polyethylene (UHMWPE) liner. A 28-mm metal head was employed in all hip prostheses.

### Anesthesia and surgery

The MDT determined the anesthesia type upon a comprehensive evaluation of each patient. Spinal anesthesia was used whenever possible [3]. General anesthesia was employed in patients with contraindications to spinal anesthesia (such as thrombocytopenia, abnormal coagulation, lumbar spondylolisthesis, *etc*.). Twenty-five patients underwent spinal anesthesia and six received general anesthesia.

**Fig. 2:**
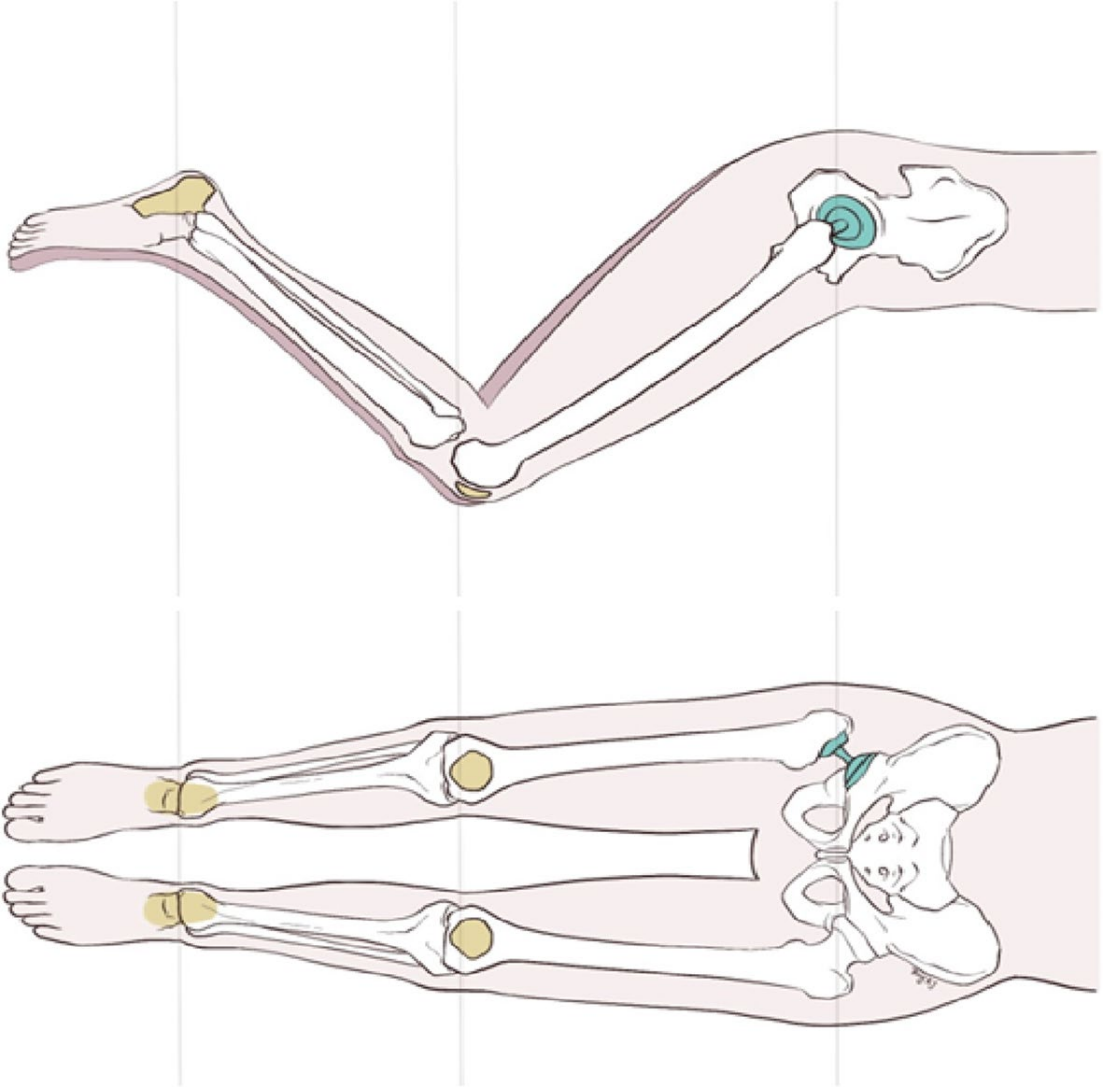
Intraoperative adjustment of lower limb length by palpation of the two poles of the patella

To shorten the surgical time and manage the fractured trochanters, all operations were performed through a posterolateral approach. All patients included in the study suffered from fractures of the greater and lesser trochanters. To gauge lower limb length, we modified the method described by Lee *et al*. [7]. Instead of intraoperative fluoroscopy, we employed direct palpation to confirm lower limb length. The patient's pelvis was shifted to a vertical position by palpating and placing both iliac crests in the same vertical plane. The operated leg was placed on the top of the contralateral leg at a similar abduction angle, and both heels were put at the same level. Then, the two poles of the patella were palpated to check lower limb length (Fig. 2). By utilizing trial components, the optimal stem size and head length were determined. If the size, stability, and leg length were satisfactory, the final stem was gently tapped into the femoral canal to achieve a meta-diaphyseal press fit. Then, the greater trochanter and the medial fracture fragments were re-attached and fixed with Kirschner wires and titanium cables. An additional greater trochanteric reattachment plate (Zimmer Biomet Corporation, Warsaw, IN, USA) was utilized if the greater trochanter was comminuted and could not be securely fixed with wires and cables alone. No drains were placed in any patients.

### Postoperative care

Patients were instructed to stand with assistance and weight-bear as tolerated with a walking aid on postoperative day 1 (POD1). Pharmacological prophylaxis for venous thromboembolism was routinely given to all patients. All patients were required to start anti-osteoporotic pharmacotherapy (Alendronate, Calcitriol plus Calcium agents, Shanghai, China) before discharge.

### Data collection

The operative time, intraoperative blood loss, rate of iatrogenic fractures, length of hospitalization, and need for blood transfusion were noted during the hospital stay. Postoperative complications, such as dislocations, deep venous thrombosis, infections, peri-prosthetic fractures, and frontal thigh pain were also recorded.

Follow-up evaluations were performed at 1, 3, 6, and 12 months and annually at the outpatient clinic. For clinical evaluations, Koval's category [14] was used for activity level and Harris hip score (HHS) was used for functional assessment. Activity levels were defined as follows: Level I, independent community ambulator; level II, community ambulator with a cane; level III, community ambulator with walker/crutches; level IV, independent household ambulator; level V, household ambulator with a cane; level VI, household ambulator with walker/crutches; and level VII, non-functional ambulator [14]. The pain was assessed on the visual analog scale (VAS). Data on radiographic outcomes including osteolysis, boney ingrowth [15], subsidence of the femoral components, lower limb length discrepancy, and heterotopic ossification were collected at each follow-up. All clinical and radiographic data were harvested by two independent research assistants.

### Statistical analysis

Statistical analysis was performed using SPSS 23.0 (IBM SPSS, Armonk, New York, NY, USA), and a *P* < 0.05 was considered statistically significant. Radiographic measurements and continuous clinical variables were assessed for significance using paired sample *t*-test, after confirming that the data followed a normal distribution. Quantitative results were reported as mean ± SD and categorical variables were presented as the median and interquartile ranges or numbers and percentages.

## Results

A total of 31 patients were followed for an average of 23 months (range, 14–26 months). One patient died 15 months after the operation due to respiratory failure. Clinical outcomes are summarized in Table 2. Mean operative time lasted 74.2 min ± 12.1 min. Mean intraoperative blood loss was 200.1 mL ± 70.2 mL. Mean hemoglobulin (Hb) on POD1 was 82.1 g/L ± 11.2 g/L, with an average decrease of 11.1 g/L ± 6.0 g/L postoperatively. The mean length of stay was 14.3 days ± 3.3 days. Of the 31 patients, 3 (9.7%) required a transfusion, with an average transfusion of 2.0 units ± 0.7 units. No iatrogenic fractures were noted during the operation.

**Table 2:** In-hospital record of patient underwent primary hemiarthroplasty

In-hospital Record	
*Intraoperative blood loss (mL)*	200.1 ± 70.2
*Hb of POD1 (g/L)*	82.1 ± 11.2
*Length of stay (days)*	14.3 ± 3.3
*Blood transfusion*	
*Number (%)*	3 (3/31, 9.7%)
*Volume (units)*	2.0 ± 0.7

*Hb* Hemoglobulin, *POD1* Postoperative Day 1

Before discharge, postoperative complications were found in 2 cases (6.5%). Thrombosis in the posterior tibial vein was identified in one patient, and he was discharged uneventfully after the placement of a vena cava filter. Another patient developed coronary heart disease and atrial fibrillation before surgery and was found to have congestive heart failure on POD4. She was discharged from the cardiovascular department after medical treatment (Spironolactone 40 mg *po bid*, Bisoprolol fumarate 5 mg *po qd*, and Aspirin 100 mg *po qd*). All the patients could ambulate independently with a walker before discharge.

The VAS score was 2.4 ± 0.8 at four weeks and 1.7 ± 0.7 at one year postoperatively, which was significantly lower than the preoperative value of 7.4 ± 1.1 (*P* < 0.05). The mean HHS was 82.1 ± 4.8 one year after surgery. According to Koval's categories, 21 patients (67.7%) regained pre-injury ambulatory status, 7 patients (22.6%) dropped 1 level of ambulatory ability, and 3 patients (3/31, 9.7%) who ambulated independently without crutches before the injury required a walker for community ambulation at the latest follow-up. Four patients (12.9%) had a limb length discrepancy ranging from 3–7 mm but without clinical symptoms. No postoperative complications were observed in any of the patients during the follow-up.

**Fig. 3:**
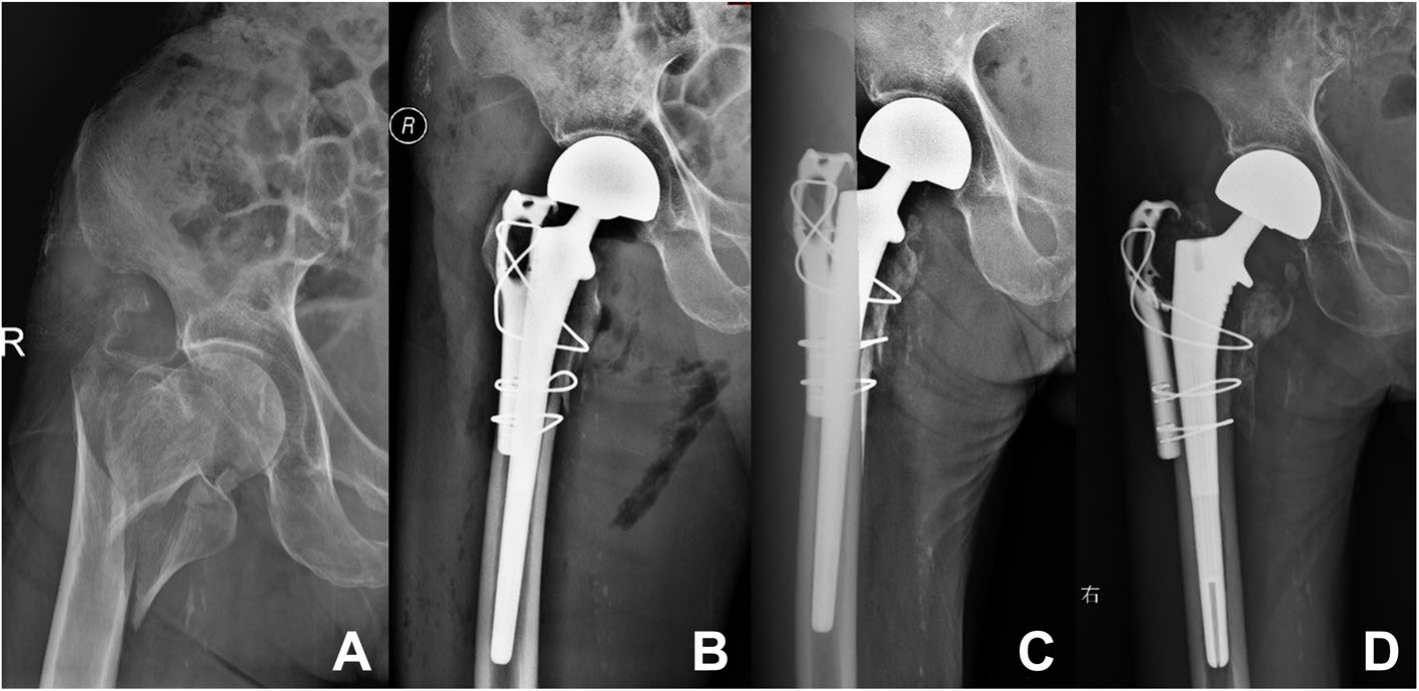
Radiographic illustrations of an 87-year-old (at the time of the surgery) male patient. **A** Pre-operative X-ray indicated unstable ITC fracture and osteoporosis; **B** Postoperative X-ray of the patient two days after the operation; **C** Follow-up X-ray of the patient three months after the operation; **D** Follow-up X-ray of the patient 18 months after the operation

All femoral stems showed radiographic evidence of bony ingrowth at the final follow-up [15]. Peri-prosthetic osteolysis and aseptic loosening were not detected in any cases. None of the patients had evidence of subsidence of femoral stem ≥ 5 mm (Fig. 3).

## Discussion

In this retrospective study, we reported the early clinical and radiographic outcomes of a cementless, bone-conserving, fully hydroxyapatite-coated revision stem used for primary hemiarthroplasty in unstable ITC fractures. Overall, the clinical and radiographic outcomes of a follow-up lasting 23 months were encouraging. Clinically, patients reported significant pain relief, were able to mobilize early, promptly returned to pre-fracture activity levels, and had an acceptably low mortality rate. Radiographically, all stems achieved bony ingrowth without evidence of subsidence or fracture. Although internal fixation with compression hip screws or proximal femoral nails is the traditional treatment for unstable ITC fracture, the results of this study demonstrated that primary hemiarthroplasty with a revision stem might also be an option for the treatment of these injuries.

Arthroplasty could potentially shorten the time to mobilization and allow patients to fully weight-bear sooner, which can notably reduce mortality in hip fractures [16, 17]. Haentjens *et al*. reported a higher incidence of pneumonia and pressure sores associated with internal fixation, possibly resulting from restriction of early weight-bearing [18]. In a study involving 230 patients, Iosifidis *et al*. found that early ambulation after hip fractures was the most significant predictive factor for long-term survival [19]. In our study, patients were allowed to fully weight-bear on POD1 and nearly 70% of them returned to their pre-injury activity level at the latest follow-up. Additionally, the mortality rate was 3.2% (1/31), considerably lower than that reported in previous literature [20]. These promising results might be attributable to the early mobilization allowed by our surgery. However, our outcome measures were limited to approximately 2 years and were tested in a small patient cohort. Furthermore, our study was also subject to selection bias, as all our patients were ASA I–III and had relatively high pre-injury activity levels, which might have contributed to the more favorable results.

It has been reported that the failure or re-operation rate of internal fixation ranges from 6% to 32% for geriatric patients with unstable ITC fractures [6]. In the present study, successful bony in-growth was achieved in all patients with no evident implant loosening or subsidence. The revision stem used in the current study was designed to fill the meta-diaphyseal junction and could attain biological fixation with proximal bone loss. So early mobilization with weight-bearing was allowed in all the patients involved. The extensive hydroxyapatite coating, which was proven to increase biological fixation and allow for even stress distribution and good long-term survival, could also enhance stability via fixation through the bone-hydroxyapatite interface [12, 13]. Additionally, guaranteed initial stability and early weight-bearing protocol would also augment osteointegration of the fracture [21]. Although long-term follow-up might witness a higher rate of implant loosening or revision, the clinical relevance could be debilitated due to lower activity levels and limited life expectancy in the elderly population.

Compared to minimally invasive internal fixation, joint arthroplasty may be associated with prolonged surgical duration and increased intraoperative blood loss [2]. With a comprehensive MDT evaluation and the use of a femoral stem already familiar to the surgeons, the average surgery time in our study was only 74.2 min ± 12.1 min, which was shorter than the time reported in similar studies [7, 8] and comparable to the time reported for internal fixation [16]. Although some investigators reported increased intraoperative blood loss in joint arthroplasty when compared to intramedullary nail fixation, our experience showed that the judicious use of tranexamic acid significantly limited blood loss. The average intraoperative blood loss was 200.1 mL ± 70.2 mL, and the average decrease of Hb was 11.1 g/L ± 6.0 g/L, with only 3 patients (9.7%, 3/31) receiving blood transfusions postoperatively. Although a recent meta-analysis reported significantly increased intraoperative blood loss in hemiarthroplasty, the authors admitted that the results had pronounced heterogeneity, and the difference might have been derived from differences in devices used and skill levels of surgeons [16]. Through a minimally invasive posterolateral approach, well-rehearsed techniques for stem implantation, and coordination of the MDT, adverse events were minimized in the present study.

A typical revision stem engages the diaphysis to achieve stability [22]. However, these conventional revision stems require aggressive reaming to the host cortical bone to properly size the femoral component and prevent subsidence. Diaphyseal reaming has been found to be associated with a high rate of perioperative femoral fractures, causes frontal thigh pain, and leads to proximal femoral stress shielding in 10–24% of cases [11, 23]. Without diaphyseal reaming, as in this study, iatrogenic bone loss is minimized. Additionally, more proximal press-fitting at the meta-diaphyseal junction of the current stem may deliver better bone loading and stress distribution. This is made evident by the fact that no iatrogenic fractures occurred intraoperatively, and no peri-prosthetic fractures were observed during the study. Furthermore, the slots in the distal portion of the stem used in the study were designed to adapt to the natural curve of the femur, and no complaints of thigh pain were lodged at any time point.

The current study is not without limitations. It was a short-term retrospective study with a small cohort, and the incidence of subsidence or loosening requires assessment over a more extended period of time. The study also suffered from the absence of a control group undergoing internal fixation, which rendered it difficult to make a direct comparison with traditional treatment modalities for these fractures.

## Conclusion

Our study exhibited that primary hemiarthroplasty using a bone-conserving, cementless, fully hydroxyapatite-coated revision stem could be a reliable alternative for the treatment of unstable ITC fractures in the geriatric population with osteoporosis. However, comprehensive preoperative assessment, individualized perioperative care, and coordination of trauma and joint surgeons are important to guarantee a favorable outcome.

## Data Availability

The datasets supporting the conclusions of this article are included within the article.
